# Mutations affecting cleavage at the p10-capsid protease cleavage site block Rous sarcoma virus replication

**DOI:** 10.1186/1742-4690-2-58

**Published:** 2005-09-27

**Authors:** Marcy L Vana, Aiping Chen, Peter Boross, Irene Weber, Dalbinder Colman, Eric Barklis, Jonathan Leis

**Affiliations:** 1 Department of Microbiology and Immunology, Feinberg School of Medicine, Northwestern University, Chicago, Illinois 60611, USA; 2 Department of Biology, Georgia State University, Atlanta, GA 30303, USA; 3 Biochemistry and Molecular Biology Department, Medical and Health Sciences Center, University of Debrecen, Debrecen, Hungary; 4 Vollum Institute and Department of Microbiology, Oregon Health and Science University, Portland, OR, 97201, USA

## Abstract

A series of amino acid substitutions (M239F, M239G, P240F, V241G) were placed in the p10-CA protease cleavage site (VVAM*PVVI) to change the rate of cleavage of the junction. The effects of these substitutions on p10-CA cleavage by RSV PR were confirmed by measuring the kinetics of cleavage of model peptide substrates containing the wild type and mutant p10-CA sites. The effects of these substitutions on processing of the Gag polyprotein were determined by labeling Gag transfected COS-1 cells with ^35^S-Met and -Cys, and immunoprecipitation of Gag and its cleavage products from the media and lysate fractions. All substitutions except M239F caused decreases in detectable Gag processing and subsequent release from cells. Several of the mutants also caused defects in production of the three CA proteins. The p10-CA mutations were subcloned into an RSV proviral vector (RCAN) and introduced into a chick embryo fibroblast cell line (DF-1). All of the mutations except M239F blocked RSV replication. In addition, the effects of the M239F and M239G substitutions on the morphology of released virus particles were examined by electron microscopy. While the M239F particles appeared similar to wild type particles, M239G particles contained cores that were large and misshapen. These results suggest that mutations affecting cleavage at the p10-CA protease cleavage site block RSV replication and can have a negative impact on virus particle morphology.

## Findings

The structural proteins of retroviruses are encoded by the *gag *gene and are translated as a single polyprotein. During or subsequent to virus budding, the Gag polyprotein is cleaved by the viral protease (PR), thereby releasing the mature structural proteins. Gag processing leads to morphological changes in the virus particle, including condensation of the capsid core, and is associated with the appearance of infectious particles [1]. It has previously been demonstrated that proper processing at several protease sites throughout RSV Gag is required for production of infectious virus [2,3]. However, the protease site separating the C-terminus of p10 and the N-terminus of CA has not been examined.

Multiple studies have highlighted the importance of cleavage at the N-terminus of retrovirus CA proteins in particle assembly and maturation. Structural studies have identified a β hairpin structure at the N-terminus of RSV CA that is thought to form after proteolysis at the p10-CA site and liberation of the N-terminus of CA [4]. Moreover, a conserved Pro residue at the extreme N-terminus of RSV CA forms a salt bridge with an internal Asp residue, thereby stabilizing the β-hairpin structure [4]. These Pro and Asp residues are highly conserved among many retrovirus CA proteins, suggesting that the β-hairpin is a common structural feature of retrovirus CA proteins [5-8]. Mutating the conserved Asp residue in HIV-1 CA (Asp51) or murine leukemia virus CA (MLV, Asp63) causes a loss in virus infectivity [8]. In addition, blocking protease cleavage at the N-terminus of MLV CA results in the production of virus that is non-infectious [9]. It has also been demonstrated that the N-terminus of CA and the residues immediately upstream of CA have a role in determining the shape of assembling retrovirus particles [8,10-13]. More specifically for RSV, it has been demonstrated that the presence of p10 on the N-terminus of CA-NC converts the *in vitro *assembly phenotype from cylindrical particles to spherical particles that resemble wild type immature RSV particles [10,11].

**Figure 1:**
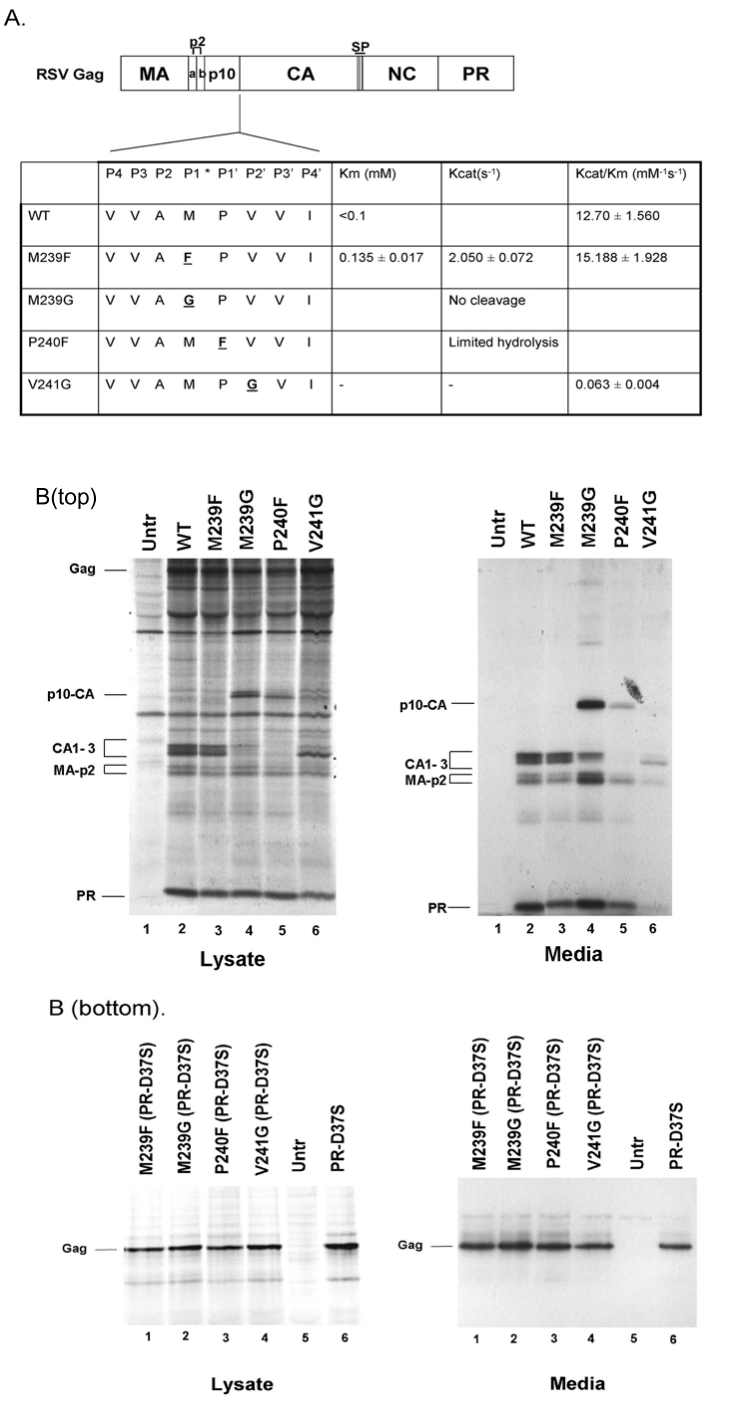
**A**. Schematic diagram of the RSV Gag polyprotein and the amino acid substitutions placed in the p10-CA protease cleavage site within Gag. The rectangle represents the RSV Gag polyprotein with the encoded protein sequences indicated by the standard nomenclature. The horizontal lines represent the PR cleavage sites. SP is the spacer peptide. The L domain of RSV Gag resides in the p2b peptide. In the box below, the P4-P1 and P1'-P4' amino acid sequence of the wild type p10-CA protease cleavage site is shown. The p10-CA mutants (underlined bold text) are shown below the wild type sequence. The results of *in vitro *protease assays examining RSV PR-mediated cleavage of peptides containing the wild type (PVVAM*PVVIKRR) and mutant p10-CA sites are also indicated. The site of p10-CA cleavage is designated with an asterisk. **B. Top**, Effect of p10-CA amino acid substitutions on processing of RSV Gag. COS-1 cells were transfected with wild type Gag or the p10-CA mutants in pSV.Myr0(HpaI). 48 hours after transfection, cells were labeled with [^35^S]-Met and Cys and Gag proteins were immunoprecipitated with an anti-RSV rabbit antiserum from the media (right panel) and lysate (left panel) fractions. Immunoprecipitated proteins were separated by SDS-PAGE and exposed to film. Lane 1, untransfected cells. Cells transfected with wild type, lane 2; M239F, lane 3; M239G, lane 4; P240F, lane 5; V241G, lane 6. **B. Bottom**. Effect of p10-CA amino acid substitutions on Gag release in the context of a protease inactivating substitution (PR-D37S). COS-1 cells were transfected and full-length Gag proteins were immunoprecipitated and separated by SDS-PAGE as above. Cells transfected with M239F/PR-D37S, lane 1; M239G/PR-D37S, lane 2; P240F/PR-D37S, lane 3; V241G/PR-D37S, lane 4; untransfected cells, lane 5; PR-D37S, lane 6.

In this study, amino acid substitutions were made in the first two N-terminal residues of CA and the last C-terminal amino acid of p10 in order to alter cleavage at the p10-CA site and examine the role of p10-CA cleavage in Gag processing and RSV replication (Fig. 1A). Previous studies focusing on the RSV NC-PR or HIV-1 MA-CA cleavage sites showed that substituting Gly at any of the P2-P2' positions resulted in greatly reduced *in vitro *hydrolysis of the peptides [14,15]. Phe substitutions of P1 provided good cleavage of the RSV NC-PR or HIV-1 MA-CA peptides, while Phe substitutions of P1' were tolerated in the RSV NC-PR peptide, but not in the HIV-1 MA-CA peptide. The ability of RSV PR to cleave peptides containing the p10-CA amino acid substitutions compared to a peptide containing the wild type p10-CA site was tested using an *in vitro *protease assay [16]. All of the substitutions except M239F led to a decrease in the rate of peptide cleavage (Fig. 1A). Substituting Phe for Met in the P1 position (M239F) had a small stimulatory effect on peptide cleavage by PR, while changing the same Met to Gly (M239G) resulted in a complete block in peptide cleavage. Similarly, changing the P1' Pro to Phe (P240F) caused a severe if not complete loss in peptide cleavage and replacement of the Val in the P2' position with Gly (V241G) resulted in a 200-fold decrease in peptide cleavage. Thus, mutating residues on either side of the cleavage junction significantly altered processing of the site.

The effects of the p10-CA substitutions on Gag processing were tested by introduction of the mutations into the context of full-length Gag and expressing the wild type or mutant Gag proteins in COS-1 cells [2,3]. Gag and its cleavage products were immunoprecipitated from the media and lysate fractions from transfected cells following metabolic labeling and were separated using SDS-PAGE (Fig. 1B, top). By comparison to wild type (Fig. 1B, top lanes 2), all of the p10-CA substitutions except M239F caused processing defects. The banding pattern in the lysate and media fractions from cells transfected with M239F (Fig. 1B, top, lanes 3) was very similar to wild type, suggesting that the M239F substitution did not affect Gag processing. In contrast, a novel and stable band representing a p10-CA fusion protein was present in the lysate and media fractions from cells transfected with the M239G (Fig. 1B, top, lanes 4) and P240F (Fig. 1B, top, lanes 5) mutants that was not present in fractions from cells transfected with wild type Gag (lanes 2 top). The presence of a p10-CA fusion indicated that these mutations resulted in a reduction in the ability of PR to cleave the p10-CA site within Gag.

In cells transfected with wild type Gag, three CA species were detected (CA1, CA2, and CA3) in the media and lysate fractions (Fig. 1B, top, lanes 2) [2,3]. These species are the result of processing of CA at its C-terminus at different sites. In contrast, in cells transfected with the M239G mutant, CA2 and CA3 were detected in the media fraction, but CA1 was not (Fig. 1B, top, lanes 4). Furthermore, mature CA proteins were not detected in the lysate. Similarly, none of the mature CA proteins were detected in the media or lysate fractions from cells transfected with the P240F (Fig. 1B, top, lanes 5) mutant, and CA1 made up the majority of the CA protein in the media and lysate fractions from cells transfected with the V241G (Fig. 1B, top, lanes 6) mutant. There also appeared to be a reduction in the amount of Gag released into the media from cells transfected with the V241G mutant compared to cells transfected with wild type Gag (Fig. 1B, top, lanes 6 and 2). This effect was most apparent when examining the signal of PR in the lysate and media fractions. The amount of PR in the lysate fraction from cells transfected with the V241G mutant was similar to wild type, but the amount of PR in the media fraction from cells transfected with the V241G mutant was greatly reduced compared to wild type. In order to determine whether the reduction in particle release observed with the V241G mutant was due to impaired Gag processing, a D37S mutation in the PR domain was constructed in the context of the p10-CA Gag mutants. COS-1 cells were transfected with the p10-CA/PR-D37S mutants and full-length Gag was immunoprecipitated from the media and lysate fractions. A similar level of Gag release was observed with all of the p10-CA/PR-D37S mutants when compared to PR-D37S (Fig. 1B, bottom), suggesting that the particle release defect observed with the V241G substitution was due to impaired Gag processing. Taken together, these results indicate that mutations to the p10-CA site of Gag affect processing of the C-terminus of CA.

**Figure 2:**
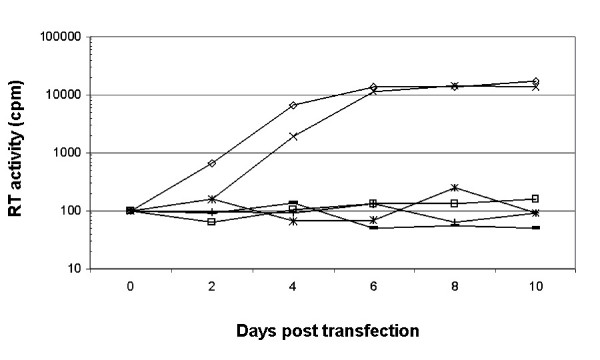
Effect of p10-CA substitutions on ability of RSV to replicate in transfected DF-1 cells. DF-1 cells were transfected with wild type RCAN, RCAN constructs containing the p10-CA mutations, or an RCAN construct containing an L domain deletion (ΔPY). At the indicated times after transfection, the RT activity in the culture medium was determined by quantification of [α-^32^P]-dTTP incorporation during reverse transcription using a polyadenylic acid (poly rA) template and a oligodeoxythymidylate (p(dT)_12–18_) primer. Wild type (◇), L-domain deletion (□), M239F (X), M239G (*), P240F (**-**), and V241G (+).

In order to determine the effects of the p10-CA substitutions on RSV replication, the p10-CA mutations were sub-cloned into the RCAN proviral vector [17]. DF-1 cells were transfected with each of the mutants, and reverse transcriptase (RT) activity was monitored in the media of transfected cells at regular intervals [18]. All of the p10-CA mutations except M239F had a detrimental effect on RSV replication (Fig. 2). The M239F mutation caused an initial delay in replication with an approximate four-fold reduction in RT activity but reached a similar peak in virus production to wild type by day six. In contrast, all of the other p10-CA mutations led to a severe block in viral replication (Fig. 2). The RT activity of these mutants could not be detected above control levels of 5TE buffer (data not shown), media from untransfected cells (data not shown), or media from cells transfected with an L domain deletion mutant (ΔPY/RCAN).

**Figure 3:**
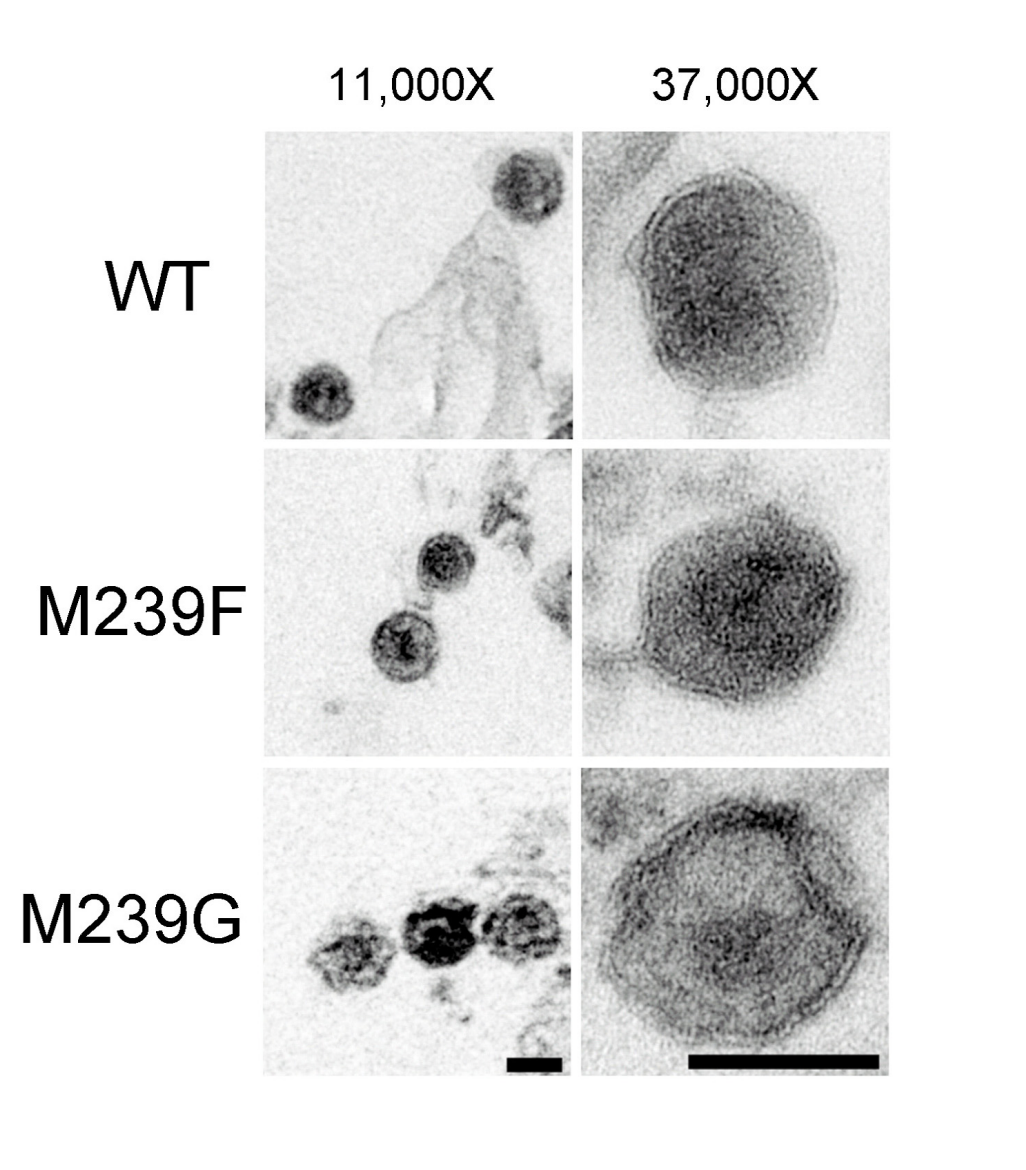
Effect of p10-CA mutations on virus particle morphology. WT (top left and right), M239F (middle left and right), and M239G (bottom left and right) viruses from transfected cells were sedimented through 20% sucrose cushions, resuspended, and processed for electron microscopy. At low magnification (left; top, middle and bottom), WT and M239F cores appeared conical or bullet-shaped, whereas M239G cores sometimes appeared conical (left-bottom, leftmost virus), but more often appeared with large misshapen cores. At higher magnification (right; top, middle and bottom), internal cores were difficult to discern without significant adjustment of image contrast levels. Size bars for the two magnifications of images appear in bottom left and right panels, and correspond to 100 nm.

To better understand the effect of the p10-CA mutations on RSV replication, wild type and p10-CA virus particles were examined using electron microscopy. Virus particles were harvested three days post transfection and viewed at a magnification of 11,000× (Fig. 3, left) and 37,000× (Fig. 3, right). We were only able to examine wild type, M239F and M239G particles by EM, as we were unable to obtain high enough amounts of P240F and V241G particles. M239F particles appeared to be similar to wild type particles in diameter (wild type; 119 ± 7 nm, MF; 118 ± 11 nm). The ratio of the cross-sectional areas of the virus core and the entire virus particle were also similar between the wild type (Fig. 3, top left and right) and M239F (Fig. 3, middle left and right) particles (wild type; 28 ± 3%, MF; 26 ± 3%). In contrast, the M239G particles (Fig. 3, bottom left and right) were larger in diameter (MG; 125 ± 5 nm) compared to the wild type and M239F particles, and had a higher ratio of core to particle cross-sectional area (MG; 45 ± 5%). It is likely that the defect in particle morphology observed with the M239G mutant played a role in the loss of replication capacity of this mutant. Together, these results highlight the importance of proper processing at the p10-CA site in RSV replication, and support previous findings demonstrating the importance of this region in retrovirus replication [4-13].

## Competing interests

The author(s) declare that they have no competing interests.

## Authors' contributions

M. L. V. constructed the p10-CA mutations, performed the Gag processing and replication assays, purified virus particles for EM analysis, and wrote the paper. A. C. constructed the D37S mutations and performed the budding assay with the D37S mutants. P. B. performed the *in vitro *protease assay. D. C. and E. B. performed the EM analysis.
